# Intermediate-High Risk Pulmonary Embolism

**DOI:** 10.1055/s-0039-3401003

**Published:** 2019-12-04

**Authors:** Rosa Mirambeaux, Francisco León, Behnood Bikdeli, Raquel Morillo, Deisy Barrios, Edwin Mercedes, Lisa Moores, Victor Tapson, Roger D. Yusen, David Jiménez

**Affiliations:** 1 Respiratory Department, Ramon y Cajal Hospital, Madrid, Spain; 2 Division of Cardiology, Department of Medicine, Columbia University Medical Center, NewYork–Presbyterian Hospital, New York, New York, United States; 3 Center for Outcomes Research and Evaluation (CORE), Yale University School of Medicine, New Haven, Connecticut, United States; 4 Cardiovascular Research Foundation (CRF), New York, New York, United States; 5 F. Edward Hebert School of Medicine, Uniformed Services University, Bethesda, Maryland, United States; 6 Pulmonary/Critical Care Division, Cedars-Sinai Medical Center, Los Angeles, California, United States; 7 Divisions of Pulmonary and Critical Care Medicine and General Medical Sciences, Washington University School of Medicine, St. Louis, Missouri, United States; 8 Medicine Department, Universidad de Alcala, Madrid, Spain; 9 CIBER de Enfermedades Respiratorias, Instituto de Salud Carlos III, Madrid, Spain

**Keywords:** pulmonary embolism, intermediate-high risk, survival, prognosis

## Abstract

Limited information exists about the prevalence, management, and outcomes of intermediate-high risk patients with acute pulmonary embolism (PE). In a prospective cohort study, we evaluated consecutive patients with intermediate-high risk PE at a large, tertiary, academic medical center between January 1, 2015 and March 31, 2019. Adjudicated outcomes included PE-related mortality and a complicated course through 30 days after initiation of PE treatment. Repeat systolic blood pressure (SBP), heart rate (HR), brain natriuretic peptide (BNP), and cardiac troponin I (cTnI) measurements, and echocardiography were performed within 48 hours after diagnosis. Among 1,015 normotensive patients with acute PE, 97 (9.6%) had intermediate-high risk PE. A 30-day complicated course and 30-day PE-related mortality occurred in 23 (24%) and 7 patients (7.2%) with intermediate-high risk PE. Seventeen (18%) intermediate-high risk patients received reperfusion therapy. Within 48 hours after initiation of anticoagulation, normalization of SBP, HR, cTnI, BNP, and echocardiography occurred in 82, 86, 78, 72, and 33% of survivors with intermediate-high risk PE who did not receive immediate thrombolysis. A complicated course between day 2 and day 30 after PE diagnosis for the patients who normalized SBP, HR, cTnI, BNP, and echocardiography measured at 48 hours occurred in 2.9, 1.4, 4.5, 3.3, and 14.3%, respectively. Intermediate-high risk PE occurs in approximately one-tenth of patients with acute symptomatic PE, and is associated with high morbidity and mortality. Normalization of HR 48 hours after diagnosis might identify a group of patients with a very low risk of deterioration during the first month of follow-up.

## Introduction

Hemodynamically unstable acute pulmonary embolism (PE) is a cardiovascular emergency, associated with high risk of death from worsening right ventricle (RV) failure and cardiogenic/obstructive shock, with an in-hospital mortality rate of > 15%. 1 2 3 For normotensive patients diagnosed with PE, risk stratification should aim to identify the group of patients deemed as having a high risk for a PE-related complicated course (intermediate-high risk PE) that might benefit from intensive monitoring or escalation of therapy. 4

Prior investigations from existing PE registries have provided some important insights into the use of prognostic tools to identify patients with intermediate-high risk PE. 5 6 According to the European Society of Cardiology (ESC) guidelines, normotensive PE patients with a positive prognostic score (i.e., Pulmonary Embolism Severity Index [PESI], simplified PESI [sPESI]), and evidence of RV dysfunction by elevated cardiac biomarkers (i.e., cardiac troponin test) and imaging should be classified into an intermediate-high risk category. 4 However, studies have shown conflicting data regarding the prognostic significance of intermediate-high risk PE. 7 8 While one study found the proportion of complications for patients in the intermediate-high risk group to be significantly higher than in the intermediate-low risk group (17.5 vs. 10%), 7 another study did not confirm these findings. 8 There remains limited contemporary information about the epidemiology, management, and outcomes of patients with intermediate-high risk PE. Further, there is uncertainty about the subgroup of patients with intermediate-high risk PE who are more likely to deteriorate, and hence may benefit from reperfusion.

Accordingly, we conducted a prospective cohort study to determine the prevalence, treatment patterns, and associated outcomes for patients with intermediate-high risk PE in routine clinical practice, and to explore the markers of early response to anticoagulant therapy.

## Methods

### Study Design

Consecutive normotensive patients with a diagnosis of acute PE between January 1, 2015 and March 31, 2019 were approached for enrollment in a prospective study. All patients provided informed consent for their participation in the study in accordance with the requirements of the ethics committee of the hospital, and the human subjects committee approved this study.

### Patients, Setting, and Eligibility Criteria

Patients were recruited from the emergency department of Ramón y Cajal Hospital, Madrid, Spain. Eligibility for this study required that patients have acute symptomatic PE confirmed by either a contrast-enhanced PE protocol helical chest computed tomography (CT), 9 a high probability ventilation–perfusion scan result according to the criteria of the Prospective Investigation of Pulmonary Embolism Diagnosis, 10 or a lower limb venous compression ultrasonography positive for a proximal deep vein thrombosis in patients with inconclusive ventilation–perfusion scans. 11

### Definition of Intermediate-High Risk PE

We defined intermediate-high risk PE as the presence of hemodynamic stability (systolic blood pressure [SBP] ≥ 90 mm Hg), a positive sPESI, and concomitant echocardiographic RV dysfunction, and positive cardiac troponin. 4

### Calculation of the sPESI

Using the baseline data collected at the time of PE diagnosis, investigators prospectively determined the sPESI. 12 The sPESI categorized patients with none of the variables present as negative, and those with at least one factor present as positive.

### Transthoracic Echocardiography

The study required that patients undergo transthoracic echocardiography within 12 hours after diagnosis of PE. Trained and certified local cardiologists interpreted each echocardiogram. The study defined echocardiographic RV dysfunction as the presence of at least two of the following: dilatation of the RV (end-diastolic diameter > 30 mm from the parasternal view or the RV appearing larger than the left ventricle from the subcostal or apical view), hypokinesis of the RV free wall (any view), and estimated systolic pulmonary artery pressure over 30 mm Hg. 13 14

### Cardiac Biomarker Determinations

The Hospital Universitario Ramon y Cajal-IRYCIS Biobank processed the biological samples. The laboratory personnel, blinded to the patients' baseline characteristics and clinical outcome, measured cardiac troponin I (cTnI) levels quantitatively by using a microparticle enzyme immunoassay (MEIA) (Abbot, United States). The study defined cTnI concentrations of > 0.05 ng/mL as indicative of myocardial injury (cTnI positive). 13 15 Brain natriuretic peptide (BNP) levels were measured by the MEIA system immunoassay in an Architect i2000 analyzer (Abbott). The study protocol defined BNP concentrations of >100 pg/mL as indicative of cardiac myocyte stretch (BNP positive). 16

### Study Endpoints and Outcome Measures

The study used PE-related mortality and a “complicated course” as the study endpoints. Investigators determined survival status by conducting patient or proxy interviews, and/or hospital chart review. Fatal PE was defined as death from PE confirmed by autopsy or death following a clinically severe PE, either initially or shortly after an objectively confirmed recurrent event, in the absence of any alternative diagnosis. A “complicated course” was defined as a composite of PE-related death, hemodynamic collapse (defined as need for cardiopulmonary resuscitation, SBP < 90 mm Hg for at least 15 minutes, need for vasopressor administration, or need for reperfusion with thrombolytic therapy or surgery), or recurrent PE within the 30 days of follow-up. Two investigators (R.M. and F.L.) adjudicated all serious adverse events.

We defined major bleeding episodes as those that required a transfusion of at least 2 units of blood, were retroperitoneal, spinal or intracranial, or were fatal.

### Treatment and Follow-Up

Between January 1, 2015 and January 1, 2018, clinicians managed patients according to their own practice (i.e., no standardization of treatment). After January 1, 2018, a Pulmonary Embolism Response Team coordinated clinical care of patients with high- and intermediate-high risk PE. The study recorded information related to patient outcomes through 30 days after the diagnosis of the acute PE.

### Statistical Analyses

The study reported categorical data as proportions and continuous data as mean ± standard deviation or median (first–third interquartile range). We used unpaired two-tailed *t* -tests or the Mann–Whitney *U* test (for those variables found not to follow a normal distribution) for comparisons in the distributions of continuous variables between intermediate-high risk versus low- and intermediate-low risk PE patients, and chi-squared or Fisher's exact tests to compare the categorical data between the two groups. We also used these tests to explore differences between the patients with intermediate-high risk PE who did and did not receive any kind of reperfusion therapies (i.e., systemic thrombolysis, local thrombolysis, percutaneous procedures, and surgical embolectomy).

For survivors who did not receive immediate (i.e., at the time of PE diagnosis) thrombolysis, repeat SBP, heart rate (HR), cTnI, and BNP measurements, and echocardiography were performed within 48 hours after initiation of anticoagulation. Assessment of vital signs was done by the study cardiologists just before the repeat echocardiogram. Normalization of prognostic tests was defined as an increase in SBP > 100 mm Hg, a decrease in HR < 100 beats per minute, a decrease in cTnI levels to ≤ 0.05 ng/mL, a decrease in BNP levels to ≤ 100 pg/mL, and absence of echocardiographic RV dilation and dysfunction. For each test risk subgroup (i.e., negative vs. positive), the proportion of patients with 30-day adverse outcomes was determined. To assess the test and performance characteristics of each test negative versus positive categories, we estimated sensitivity, specificity, and positive and negative predictive values.

Analyses were performed using SPSS, version 23.0 for the personal computer (SPSS Inc., Chicago, Illinois, United States). All hypothesis tests were two-sided, with a significance level of 0.05.

## Results

**Fig. 1:**
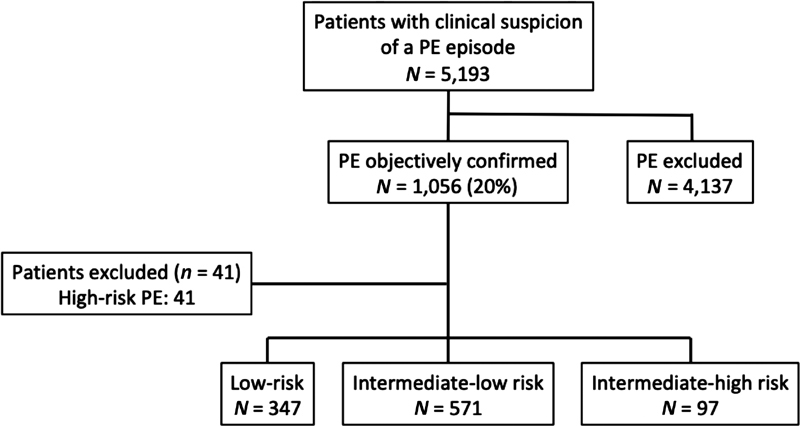
Patient flow diagram. PE, pulmonary embolism.

We enrolled 1,015 normotensive patients with acute PE (486 men and 529 women) from January 1, 2015 to March 31, 2019 ( Fig. 1 ). Overall, 97 patients (9.6%; 95% confidence interval [CI], 7.8–11.5%) had intermediate-high risk, 571 (56%) patients had intermediate-low risk, and 347 (34%) had low-risk PE. Patients with intermediate-high risk PE differed significantly from those with low- and intermediate-low risk PE in preexisting medical conditions, and in relevant clinical, physiologic, and laboratory parameters. As expected, patients with intermediate-high risk PE were older and had more comorbid diseases (immobility, high-risk sPESI), and signs of clinical severity (tachycardia, hypoxemia, and hypotension), compared with those with low- or intermediate-low risk PE ( Table 1 ).

**Table 1:** Patient characteristics ( *n* = 1,015)

	Intermediate-high risk PE *N* = 97	Low- or intermediate-low risk PE *N* = 918	*p* -Value
Clinical characteristics,			
Age, y (mean ± SD)	70.4 ± 16.2	66.8 ± 16.3	0.04
Age > 80 y	28 (30)	166 (18)	0.01
Male gender	43 (44)	443 (48)	0.52
Risk factors for VTE,			
Cancer a	7 (7.2)	108 (12)	0.24
Recent surgery b	7 (7.2)	102 (11)	0.24
History of VTE	8 (8.2)	180 (20)	< 0.01
Immobilization c	22 (37)	211 (23)	< 0.01
Comorbid diseases			
Chronic lung disease	10 (11)	93 (11)	1.00
Chronic heart disease	5 (5.2)	39 (4.2)	0.60
Clinical symptoms and signs at presentation			
Syncope	29 (33)	138 (15)	< 0.001
Chest pain	43 (36)	413 (45)	0.11
Dyspnea	76 (85)	771 (84)	1.00
Heart rate ≥ 110/min	42 (43)	205 (22)	< 0.001
Arterial oxyhemoglobin saturation (SaO _2_ ) < 90%	57 (59)	147 (26)	< 0.001
SBP < 100 mm Hg	8 (8.2)	38 (4.1)	0.07
sPESI 12			
Low-risk	0 (0)	347 (38)	<0.001
High-risk	97 (100)	571 (62)	< 0.001
**Echocardiography and cardiac biomarkers, n (%)**			
RV dysfunction	97 (100)	29/242 12	<0.001
cTnI > 0 ng/mL	97 (100)	115/564 20	<0.001

Abbreviations: cTnI, cardiac troponin I; PE, pulmonary embolism; RV, right ventricle; SBP, systolic blood pressure; SD, standard deviation; sPESI, simplified Pulmonary Embolism Severity Index; VTE, venous thromboembolism.

a Active or under treatment in the last year.

b In the previous month.

c Immobilized patients defined as nonsurgical patients with limited mobility (i.e., total bed rest with bathroom privileges) for ≥ 4 days in the month prior to PE diagnosis.

### Treatment Patterns

Seventeen (18%; 95% CI, 11–27%) intermediate-high risk patients received reperfusion therapy (6 patients received immediate thrombolysis at the time of PE diagnosis, and 11 patients after clinical deterioration). No patient received surgical or catheter embolectomy. Patients who received reperfusion were younger than patients who did not receive reperfusion (57 ± 20 vs. 73 ± 14 years), and more frequently presented with chest pain, tachycardia, and hypotension, compared with intermediate-high risk patients who did not receive reperfusion ( Table 2 ). Interestingly, the two treatment groups had similar proportions of male gender, history of heart failure, chronic obstructive pulmonary disease (COPD), cancer, recent surgery, history of venous thromboembolism, and immobilization.

**Table 2:** Clinical characteristics of patients with intermediate-high risk PE ( *n* = 97) who did or did not receive thrombolysis

	Received thrombolysis *N* = 17	Did not receive thrombolysis *N* = 80	*p* -Value
Clinical characteristics,			
Age, y (mean ± SD)	56.6 ± 20.3	73.3 ± 13.7	< 0.001
Age > 80 y	0 (0)	28 (35)	< 0.01
Male gender	8 (47)	35 (44)	1.00
Risk factors for VTE,			
Cancer a	1 (5.9)	6 (7.5)	1.00
Recent surgery b	1 (5.9)	6 (7.5)	1.00
History of VTE	2 (12)	6 (7.5)	0.63
Immobilization c	3 (18)	19 (24)	0.75
Comorbid diseases			
Chronic lung disease	0 (0)	10 (13)	0.20
Chronic heart disease	0 (0)	5 (6.3)	0.58
Clinical symptoms and signs at presentation			
Syncope	4 (24)	25 (31)	0.77
Chest pain	12 (71)	31 (39)	0.03
Dyspnea	13 (76)	63 (79)	1.00
Heart rate ≥ 110/min	13 (76)	29 (36)	0.02
Arterial oxyhemoglobin saturation (SaO _2_ ) < 90%	7 (41)	50 (63)	0.17
SBP < 100 mm Hg	5 (29)	3 (3.8)	< 0.01

Abbreviations: PE, pulmonary embolism; SBP, systolic blood pressure; SD, standard deviation; VTE, venous thromboembolism.

a Active or under treatment in the last year.

b In the previous month.

c Immobilized patients defined as nonsurgical patients with limited mobility (i.e., total bed rest with bathroom privileges) for ≥ 4 days in the month prior to PE diagnosis.

### Outcomes

Outcomes data were available for all patients through the 30-day study follow-up. The 30-day all-cause mortality rates were 10% (95% CI, 5.1–18%) in patients with intermediate-high versus 4.0% (95% CI, 2.9–5.6%) in those with low- and intermediate-low risk PE. In the intermediate-high risk group, most deaths (7 of 10 deaths; 70%; 95% CI, 35–93%) were attributable to PE, while 3 patients (3.1%; 95% CI, 0.6–8.8%) died from other causes (cancer 2, and COPD 1). Twenty-three of 97 intermediate-high risk PE patients (24%; 95% CI, 16–33%) had a complicated course. In addition to the 7 PE-related deaths, complicated course was due to nonfatal hemodynamic collapse deterioration in 16 patients ( Supplementary Table S1 ). Two of 97 patients (2.1%; 95% CI, 0.3–7.3%) had recurrent symptomatic PE (both fatal), and 4 patients (4.1%; 95% CI, 1.1–10%) suffered a major bleeding episode (retroperitoneal 2, intracranial 1, and need for transfusion 1).

In the subgroup of patients with intermediate-high risk PE, PE-related mortality within 30 day of PE diagnosis occurred in 4 patients (3.2%; 95% CI, 0.9–8.1%) who received delayed reperfusion (i.e., after clinical deterioration), and in 3 patients (3.8%; 95% CI, 0.8–10.6%) who did not receive reperfusion. Of the patients who received thrombolysis, 5.9% (1 of 17 patients) had recurrent PE during the 30-day study follow-up period. Of those who did not receive thrombolysis, 1.3% (1 of 80 patients) recurred during follow-up. Of the patients who received thrombolysis, 12% (2 of 17 patients) bled during the 30-day study follow-up period. Of those who did not receive thrombolysis, 2.5% (2 of 80 patients) bled during follow-up.

### Response to Anticoagulation

Within 48 hours after initiation of anticoagulation, repeat SBP, HR, cTnI, and BNP measurements, and echocardiography were obtained in 85 survivors who did not receive immediate thrombolysis. Of these, 11 (13%; 95% CI, 6.6–22%) experienced a complicated course and 8 (9.4%; 95% CI, 4.2–18%) died between day 2 and day 30 after PE diagnosis. Overall, 5 patients (5.9%) died from definite or possible PE, 1 (1.2%) from COPD, and 2 (2.4%) from cancer. One patient had an episode of (fatal) recurrent PE, and 2 patients had an episode of nonfatal major bleeding. Normalization of SBP, HR, cTnI, BNP, and echocardiography occurred in 82, 86, 78, 72, and 33%, respectively ( Table 3 ).

**Table 3:** Characteristics of tests performed 48 hours after PE diagnosis for predicting 30-day adverse events

		Systolic blood pressure normalization	Heart rate normalization	Cardiac troponin I normalization	Brain natriuretic peptide normalization	Echocardiography normalization
Measurement normalization, % (95% CI)		82 (73–90)	86 (77–92)	78 (67–86)	72 (61–81)	33 (23–44)
Sensitivity, % (95% CI)	Complicated course	82 (48–97)	91 (57–100)	73 (39–93)	82 (48–97)	64 (32–88)
	All-cause mortality	75 (36–96)	100	63 (26–90)	75 (36–96)	75 (36–96)
	PE-related mortality	60 (17–93)	100	80 (30–99)	80 (30–99)	100
Specificity, % (95% CI)	Complicated course	92 (83–97)	97 (90–100)	85 (75–92)	80 (68–88)	32 (22–44)
	All-cause mortality	88 (78–94)	95 (87–98)	82 (71–89)	77 (65–85)	34 (24–46)
	PE-related mortality	85 (75–92)	91 (82–96)	81 (71–89)	75 (64–84)	35 (25–47)
Positive predictive value, % (95% CI)	Complicated course	60 (33–83)	83 (51–97)	42 (21–66)	38 (20–59)	12 85.5–24)
	All-cause mortality	40 (17–67)	67 (35–89)	26 (10–51)	25 (11–47)	11 (4.4–22)
	PE-related mortality	20 (5.3–49)	42 (17–71)	21 (7.0–46)	17 (5.5–38)	8.8 (3.3–20)
Negative predictive value, % (95% CI)	Complicated course	97 (89–100)	99 (92–100)	95 (86–99)	97 (88–99)	86 (66–95)
	All-cause mortality	97 (89–100)	100	95 (86–99)	97 (88–99)	93 (75–99)
	PE-related mortality	97 (89–100)	100	98 (91–100)	98 (90–100)	100
Positive likelihood ratio (95% CI)	Complicated course	10 (4.5–23)	34 (8.5–134)	4.9 (2.5–9.4)	4.0 (2.4–6.9)	0.9 (0.6–1.5)
	All-cause mortality	6.4 (3.1–13)	19 (7.4–50)	3.4 (1.7–7.0)	3.2 (1.8–5.7)	1.1 (0.7–1.7)
	PE-related mortality	4.0 (1.7–9.7)	11 (5.6–23)	4.3 (2.3–8.0)	3.2 (1.8–5.7)	1.5 (1.3–1.8)
Negative likelihood ratio (95% CI)	Complicated course	0.2 (0.1–0.7)	0.1 (0.0–0.6)	0.3 (0.1–0.8)	0.2 (0.1–0.8)	1.1 (0.5–2.6)
	All-cause mortality	0.3 (0.1–0.9)	0	0.5 (0.2–1.1)	0.3 (0.1–1.1)	0.7 (0.2–2.6)
	PE-related mortality	0.5 (0.2–1.4)	0	0.3 (0.0–1.4)	0.3 (0.1–1.6)	0

Abbreviations: CI, confidence interval; PE, pulmonary embolism.

For the patients who normalized SBP, HR, cTnI, BNP, and echocardiography measured at 48 hours, a complicated course occurred in 2.9, 1.4, 4.5, 3.3, and 14.3%, respectively ( Table 3 ). The sensitivity, specificity, and predictive values for the tests performed at 48 hours for predicting 30-day adverse events are listed in Table 3 .

## Discussion

In this prospective cohort study, intermediate-high risk PE occurred in 10% of normotensive patients with acute symptomatic PE, with markedly worse outcomes compared with those with low- or intermediate-low risk PE. During the first 30 days of follow-up, a complicated course occurred in one-fourth of the patients, and 30-day PE-related mortality rate was approximately 7%. Only one-fifth of the patients with intermediate-high risk PE received thrombolytic therapy, most of them after clinical deterioration. Normalization of clinical markers, including HR within 48 hours of PE diagnosis, identified patients with an uncomplicated course during the first month of anticoagulant therapy.

Identification of intermediate-high risk PE has evolved over time. Studies of patients with normotensive PE found that those with echocardiographic RV dysfunction and myocardial injury had a greater risk of short-term death compared with patients with either echocardiographic RV dysfunction or elevated troponin levels (or none). 17 18 Accordingly, the ESC guidelines defined intermediate-high risk patients with acute symptomatic PE as those who are hemodynamically stable, and have myocardial injury and RV dysfunction. 4 More recent observational studies have suggested an incremental prognostic value of the association of markers of RV dysfunction and injury with clinical variables. 6 19 The prevalence of intermediate-high risk in the study cohort (10%) was lower than in a previous study in which the prevalence was 30%. 8 This discrepancy may be explained at least in part because the present study used the combination of a positive sPESI with echocardiographic RV dysfunction and myocardial injury for identifying the more-severe intermediate-risk patients with acute PE, as recommended by the 2014 and 2019 ESC guidelines. 4 20

The Pulmonary Embolism Thrombolysis (PEITHO) trial was a randomized, double-blind trial that assessed the efficacy and safety of tenecteplase in normotensive patients with RV dysfunction/enlargement on echocardiography or CT, as well as myocardial injury as indicated by a positive test for cTnI or troponin T. 21 Compared with the 10% 30-day mortality in our study cohort, short-term death occurred infrequently in the trial's placebo arm (3.2%). This finding might support the requirement of clinical variables in addition to cardiac biomarkers and imaging testing to identify the sickest normotensive patients with acute symptomatic PE.

Practice guidelines suggest that most patients with intermediate-high risk PE should receive standard anticoagulation alone, 4 22 and guideline-adherent management strongly correlates with patient outcomes. 23 In this study, only a small proportion of patients received immediate thrombolytic therapy. Since these patients had more severe hemodynamic decompensation compared with those who received delayed thrombolysis, future trials should evaluate the efficacy and safety of reperfusion therapies in intermediate-high risk patients who have an overwhelming accumulation of prognostic factors indicative of a more marked cardiopulmonary impairment.

Clinical practice guidelines suggest close monitoring of patients with intermediate-high risk PE, and prompt treatment if decompensation occurs. However, it is not clear how long these patients should be monitored, and the optimal method to assess early response to anticoagulant therapy. In the PEITHO trial, mean time from enrolment to deterioration in the placebo arm was 1.8 ± 1.6 days. 21 For this reason, this study assessed clinical variables, cardiac biomarkers, and echocardiography within 48 hours after diagnosis of acute PE to explore the optimal method to assess response to anticoagulant treatment. Our results suggest that resolution of tachycardia might be a reliable tool to identify those patients with a negligible risk of deterioration within the first few days. Compared with cardiac biomarker testing and echocardiography, measurement of HR is a simple, inexpensive, rapid, repeatable, and easily interpretable tool for risk assessment.

Our findings should be interpreted in the context of our study design and its limitations. Despite the large number of normotensive patients with acute PE assessed for this study, the number of patients with intermediate-high risk PE did not allow for more precision in our estimates. In the present study, echocardiograms were done and interpreted by a limited number of certified cardiologists and thus the results cannot be necessarily applied to less experienced operators. The study protocol did not collect information on clot location and degree of obstruction in a standardized fashion. Thus, the study cannot report such information. Further, this was a single-center study at a large referral center and treatment-related decisions may not be necessarily reflective of all other centers.

In conclusion, in this cohort of consecutive patients with acute PE, intermediate-high risk PE was found in 10% of normotensive patients. Such patients had markedly worse outcomes compared with other normotensive patients with PE, suggesting the need for close monitoring. Assessment of HR may be useful to estimate the response to anticoagulation and the risk of deterioration.

## References

[1] Kucher N Rossi E De Rosa M Goldhaber S Z Massive pulmonary embolism Circulation 2006 113 04 577 582 16432055 10.1161/CIRCULATIONAHA.105.592592

[2] Goldhaber S Z Visani L De Rosa M Acute pulmonary embolism: clinical outcomes in the International Cooperative Pulmonary Embolism Registry (ICOPER) Lancet 1999 353(9162):1386 1389 10227218 10.1016/s0140-6736(98)07534-5

[3] Jiménez D Bikdeli B Barrios D Epidemiology, patterns of care and mortality for patients with hemodynamically unstable acute symptomatic pulmonary embolism Int J Cardiol 2018 269 327 333 30025658 10.1016/j.ijcard.2018.07.059

[4] Konstantinides S V Torbicki A Agnelli G 2014 ESC guidelines on the diagnosis and management of acute pulmonary embolism Eur Heart J 2014 35 43 3033 3069, 3069a–3069k 25173341 10.1093/eurheartj/ehu283

[5] Jiménez D Aujesky D Moores L Combinations of prognostic tools for identification of high-risk normotensive patients with acute symptomatic pulmonary embolism Thorax 2011 66 01 75 81 20978032 10.1136/thx.2010.150656

[6] Bova C Sanchez O Prandoni P Identification of intermediate-risk patients with acute symptomatic pulmonary embolism Eur Respir J 2014 44 03 694 703 24696111 10.1183/09031936.00006114

[7] Jimenez D Lobo J L Fernandez-Golfin C Effectiveness of prognosticating pulmonary embolism using the ESC algorithm and the Bova score Thromb Haemost 2016 115 04 827 834 26738514 10.1160/TH15-09-0761

[8] Becattini C Agnelli G Lankeit M Acute pulmonary embolism: mortality prediction by the 2014 European Society of Cardiology risk stratification model Eur Respir J 2016 48 03 780 786 27174887 10.1183/13993003.00024-2016

[9] Remy-Jardin M Remy J Wattinne L Giraud F Central pulmonary thromboembolism: diagnosis with spiral volumetric CT with the single-breath-hold technique--comparison with pulmonary angiography Radiology 1992 185 02 381 387 1410342 10.1148/radiology.185.2.1410342

[10] PIOPED Investigators.Value of the ventilation/perfusion scan in acute pulmonary embolism. Results of the prospective investigation of pulmonary embolism diagnosis (PIOPED) JAMA 1990 263 20 2753 2759 2332918 10.1001/jama.1990.03440200057023

[11] Kearon C Ginsberg J S Hirsh J The role of venous ultrasonography in the diagnosis of suspected deep venous thrombosis and pulmonary embolism Ann Intern Med 1998 129 12 1044 1049 9867760 10.7326/0003-4819-129-12-199812150-00009

[12] Jiménez D Aujesky D Moores L Simplification of the Pulmonary Embolism Severity Index for prognostication in patients with acute symptomatic pulmonary embolism Arch Intern Med 2010 170 15 1383 1389 20696966 10.1001/archinternmed.2010.199

[13] Jiménez D Kopecna D Tapson V Derivation and validation of multimarker prognostication for normotensive patients with acute symptomatic pulmonary embolism Am J Respir Crit Care Med 2014 189 06 718 726 24471575 10.1164/rccm.201311-2040OC

[14] Grifoni S Olivotto I Cecchini P Short-term clinical outcome of patients with acute pulmonary embolism, normal blood pressure, and echocardiographic right ventricular dysfunction Circulation 2000 101 24 2817 2822 10859287 10.1161/01.cir.101.24.2817

[15] Jiménez D Díaz G Molina J Troponin I and risk stratification of patients with acute nonmassive pulmonary embolism Eur Respir J 2008 31 04 847 853 18094010 10.1183/09031936.00113307

[16] Pieralli F Olivotto I Vanni S Usefulness of bedside testing for brain natriuretic peptide to identify right ventricular dysfunction and outcome in normotensive patients with acute pulmonary embolism Am J Cardiol 2006 97 09 1386 1390 16635617 10.1016/j.amjcard.2005.11.075

[17] Scridon T Scridon C Skali H Alvarez A Goldhaber S Z Solomon S D Prognostic significance of troponin elevation and right ventricular enlargement in acute pulmonary embolism Am J Cardiol 2005 96 02 303 305 16018861 10.1016/j.amjcard.2005.03.062

[18] Binder L Pieske B Olschewski M N-terminal pro-brain natriuretic peptide or troponin testing followed by echocardiography for risk stratification of acute pulmonary embolism Circulation 2005 112 11 1573 1579 16144990 10.1161/CIRCULATIONAHA.105.552216

[19] Dellas C Tschepe M Seeber V A novel H-FABP assay and a fast prognostic score for risk assessment of normotensive pulmonary embolism Thromb Haemost 2014 111 05 996 1003 24477222 10.1160/TH13-08-0663

[20] Konstantinides S V Meyer G Becattini C 2019 ESC Guidelines for the diagnosis and management of acute pulmonary embolism developed in collaboration with the European Respiratory Society (ERS)Eur Heart J2019Doi: 10.1093/eurheartj/ehz405 [Epub ahead of print] 10.1093/eurheartj/ehz405 31504429

[21] Meyer G Vicaut E Danays T Fibrinolysis for patients with intermediate-risk pulmonary embolism N Engl J Med 2014 370 15 1402 1411 24716681 10.1056/NEJMoa1302097

[22] Kearon C Akl E A Ornelas J Antithrombotic therapy for VTE disease: Chest guideline and expert panel report Chest 2016 149 02 315 352 26867832 10.1016/j.chest.2015.11.026

[23] Jiménez D Bikdeli B Barrios D Management appropriateness and outcomes of patients with acute pulmonary embolism Eur Respir J 2018 51 05 1.800445E6 10.1183/13993003.00445-2018 29724918

