# Supplementary material for: Intermediate-High Risk Pulmonary Embolism
Source: TH Open. 2019 Dec 4;3(4):e356–63. doi: 10.1055/s-0039-3401003 (PMC6892655; doi:10.1055/s-0039-3401003)
Supplement: Supplementary file 1 — Supplementary Material [file 10-1055-s-0039-3401003-s190048oa.pdf]

Supplementary Table S1 30-day clinical events after diagnosis and treatment for hemodynamically stable patients with acute symptomatic pulmonary embolism

	Intermediate-high risk PE<br>$N = 97$	Low- or intermediate-low risk PE<br>$N = 918$	*p*-Value
**Primary outcome, *n* (%)**			
All-cause death	10 (10%)	23 (2.5%)	<0.001
PE-related death	7 (7.2%)	11 (1.2%)	<0.001
**Secondary outcomes, *n* (%)**			
Complicated course[a]	23 (24%)	19 (2.1%)	<0.001
Hemodynamic collapse[b]	17 (18%)	16 (1.7%)	<0.001
Recurrent PE	2 (2.1%)	4 (0.4%)	0.11
Nonfatal recurrent PE	0 (0%)	3 (0.3%)	1.00
Major bleeding	4 (4.1%)	33 (3.6%)	0.77

Abbreviation: PE, pulmonary embolism.
[a]Defined as PE-related mortality, or hemodynamic collapse, or recurrent PE.
[b]Defined as the use of at least one of the following: cardiopulmonary resuscitation, systolic blood pressure < 90 mm Hg for at least 15 minutes, need for catecholamine administration, or need for thrombolysis.
